# Measurements of chlorinated volatile organic compounds emitted from office printers and photocopiers

**DOI:** 10.1007/s11356-014-3672-3

**Published:** 2014-10-18

**Authors:** Joanna Kowalska, Małgorzata Szewczyńska, Małgorzata Pośniak

**Affiliations:** Department of Chemical, Aerosol and Biological Hazards, Central Institute for Labour Protection — National Research Institute, Czerniakowska 16, 00-701 Warsaw, Poland

**Keywords:** Chlorinated compounds, VOCs, Emission, Test chamber, Thermal desorption, GC/MS, Office printers, Copiers

## Abstract

Office devices can release volatile organic compounds (VOCs) partly generated by toners and inks, as well as particles of paper. The aim of the presented study is to identify indoor emissions of volatile halogenated organic compounds into the office workspace environment. Mixtures of organic pollutants emitted by seven office devices, i.e. printers and copiers, were analyzed by taking samples in laboratory conditions during the operation of these appliances. Tests of volatile organic compound emissions from selected office devices were conducted in a simulated environment (test chamber). Samples of VOCs were collected using three-layered thermal desorption tubes. Separation and identification of organic pollutant emissions were made using thermal desorption combined with gas chromatography coupled to mass spectrometry. Test chamber studies indicated that operation of the office printer and copier would contribute to the significant concentration level of VOCs in typical office indoor air. Among the determined volatile halogenated compounds, only chlorinated organic compounds were identified, inter alia: trichloroethylene — carcinogenic — and tetrachloroethylene — possibly carcinogenic to human. The results show that daily exposure of an office worker to chemical factors released by the tested printing and copying units can be variable in terms of concentrations of VOCs. The highest emissions in the test chamber during printing were measured for ethylbenzene up to 41.3 μg m^−3^, xylenes up to 40.5 μg m^−3^ and in case of halogenated compounds the highest concentration for chlorobenzene was 6.48 μg m^−3^. The study included the comparison of chamber concentrations and unit-specific emission rates of selected VOCs and the identified halogenated compounds. The highest amount of total VOCs was emitted while copying with device D and was rated above 1235 μg m^−3^ and 8400 μg unit^−1^ h^−1^ on average.

**Electronic supplementary material:**

The online version of this article (doi:10.1007/s11356-014-3672-3) contains supplementary material, which is available to authorized users.

## Introduction

Increasing number of office workers and those responsible for health and safety conditions in workplaces realize that spending time in modern, well-equipped offices can adversely affect health. The cause of typical allergic symptoms such as inflammation of mucous membranes, i.e., bronchial asthma, chronic laryngitis and bronchitis, is the most common exposure to harmful factors, including multi-component mixtures of chemical substances in the air. Prolonged exposure to certain substances can cause long-time effects in the form of respiratory and circulatory system diseases and even cancer. The results of studies conducted worldwide in various centres pay special attention to the cleanliness of the environment in which people live from an early age (pre-school) in which they work (workplaces, non-industrial workplaces) and rest (houses, flats and outside air).

Office work environment is a place where a group of employees spend large part of the day in closed rooms. Therefore, in order to improve indoor air quality, it is necessary to study different emission sources of chemical compounds. Construction materials, furniture, office equipment and polluted outdoor air all form a group of potential sources of volatile organic compounds (VOCs) that exist in the office environment (Zabiegała 2006; Król et al. 2011; Han et al. 2012). In order to assess the impact of potential emission sources, measurements of individual volatile organic compounds (VOCs) are performed specific to the type of materials. The most frequently determined compounds emitted from office devices include aromatics — benzene, toluene, xylenes, as well as other benzene homologues, aliphatic hydrocarbons, esters and aldehydes (Smola et al. 2002; Kagi et al. 2007; Wang et al. 2011; Wilke et al. 2009).

Pioneering studies in this area concerning chemical compounds emitted from office machines were carried out in the 1990s of the 20th century (Wolkoff 1990; Wolkoff et al. 1992, 1993; Leovic et al. 1996; Brown 1999). Office devices can release VOCs partly generated by toners and inks that are subject to heating during the printing process, as well as particles of paper. Air emissions may include ozone, nitrogen oxides, VOCs, aldehydes, polycyclic aromatic compounds and ultrafine particles (Brown 1999; Tuomi et al. 2000; Gminski and Mersch-Sundermann 2006; Tang et al. 2012; Schripp et al. 2009). Toners used in copiers usually contain resin: a copolymer of styrene and acrylates (up to 55 %), iron oxides as pigment (up to 50 %) and up to 3 % of amorphous silica—a supplementary anti-caking agent. The resin, when subjected to heat, fixes (adheres) the pigment particles to the paper. The toner particles, which have mean aerodynamic diameter of 6–8 μm (Kim et al. 2009; Ewers and Nowak 2006), facilitate deep penetration into the human respiratory system. The paper fed into the office copiers is also a source of indoor emissions of very fine aerosol particles (Wensing et al. 2008; Gminski and Mersch-Sundermann 2006).

The referenced studies indicate that toner heated to temperature levels found inside copiers releases styrene, xylenes, ethylbenzene, acetophenone, benzaldehyde and many other benzene derivatives (Henschel et al. 2001). Some of these compounds are pollutants derived from styrene/acrylate polymers, e.g. unreacted monomers of styrene or ethylbenzene which are used in styrene production. The toner manufacturing process may also generate compound oxidation side products such as acetophenone, benzaldehyde, benzoic acid and phenol (Henschel et al. 2001). Usually, their concentrations are low (at ppb level); however, some of them are still toxic and/or carcinogenic (Gminski and Mersch-Sundermann 2006). Despite the low concentration of toxic pollutants, they are dangerous to people working in offices due to long-term exposure (Bakò-Birò et al. 2004; Salonen et al. 2009). The poor quality of indoor air can adversely affect human health. Specific health effect is often claimed for individual pollutant (e.g. benzene, tetrachloroethylene and trichloroethylene increase the risk of cancer). The mixture of VOCs in indoor air is often held responsible for irritation symptoms, respiratory illness, headache, fatigue defined as sick building syndrome (SBS) or building-related illness (BRI).

Halogenated organic compounds, despite harmful effects on the human body, are rarely identified and analyzed among the VOCs which are emitted from the office equipment items since their concentrations are very low (Lee et al. 2001; Wilke et al. 2009). The aforementioned compounds are however determined in indoor rooms, including offices (Zuraimi et al. 2006; Hsieh et al. 2006; Bruno et al. 2008). Ongwandee and co-authors (2011) measured the quality of 17 air-conditioned office buildings in Bangkok. There were five chlorinated aliphatic hydrocarbons among the 13 identified VOCs, i.e. tetrachloroethene, trichloroethene, 1,2-dichloroethene, 1,2-dichloropropane and chloroform (trichloroethene). Ongwandee and co-authors (2011) explained the presence of chloroorganic compounds identified in the office air with the emission of dry-cleaned clothes, the use of chemical substances used for cleaning the carpets or furniture, correction fluids or other solvents.

Identification of chemical sources in the office environment that induces or intensifies these health effects is complicated due to the presence of various possible emission sources. Emission of chemicals in an indoor environment can result from the materials used in indoor finishing or in the final products and can be tested in test chambers or test cells (Schripp et al. 2007; Bakò-Birò et al. 2004; Yu et al. 2011; Wang et al. 2011; Marć and Namieśnik 2014). Emission assessments of office devices are carried out using test chambers that have sufficiently large dimensions to hold a final product with a volume of 1 or 5 m^3^ or even above 50 m^3^ at pre-set air temperatures and humidity (Kirkeskov et al. 2009; Schripp et al. 2007, 2009; Makowski and Ohlmeyer 2006; Katsoyiannis et al. 2008).

The subject of this study is the analysis of air in the test chamber in order to measure volatile organic compounds released during the operation of office inkjet printers, laser printers and copiers. Specifically, the authors focused on identifying and determining halogenated VOCs and the indication of emission sources for those compounds using an analytical method of thermal desorption combined with gas chromatography coupled to mass spectrometry (TD/GC-MS).

## Experiment

### Chemicals and standards

A standard solution of 40 VOCs in methanol (200 μg/mL) (EPA 524.2 VOC mix, Supelco) was used for the qualitative analysis. The solvent methanol (reagent grade, Merck KGaA) was used. Working gas standard mixtures were prepared by diluting 5 μl of standard solution in nitrogen (99.999 % purity, Multax s.c.) in a 6-L steel canister (Silcocan, Restek). Finally, the canister was made up with nitrogen with a pressure of about 0.2 MPa. The content of each analyte was equal to 0.083 ng in 1 mL of the prepared gas mixture.

### Tested office devices

Tests were carried out with the use of the following selection of common and generally available devices present in an office workspace:

- Office printers:
  - Laser printer, year of manufacture (YOM): 1998, black print (device A)
  - Laser printer, YOM: 2007, colour print (device B)
  - Inkjet printer, YOM: 2003, colour print (device C)
- Copying devices:
  - Photocopier, YOM: 2007 (device D)
  - Photocopier, YOM: 2011 (device E)
  - Photocopier, YOM: 2003 (device F)
  - Multi-function device (MFD) used in the office for copying, printing, faxing and scanning purposes, YOM: 2004 (device G)

A white A4-sized paper weighing 80 g m^−2^and original toners recommended by the devices’ manufacturers (company toners) were used for printing and copying. During the tests, each office device was operated using the black and white mode of simplex printing and copying. The printed part covered c.a. 75 % of each page.

### Air sampling

To separate the printers and photocopiers from external agents, they were placed in a closable measuring chamber made of PLEXIGLAS® panels (with dimensions of 68 cm × 60 cm × 67 cm). Printing and copying devices subjected to testing as well as samplers (three-layered thermal desorption tubes) connected with suction apparatus were placed in the chamber. For quantitative tests, air samples were taken during the operation of selected office printing and photocopying devices at room temperature of 20–25 °C and relative ambient humidity of 40–50 %.

For identification and quantification, known volumes of chamber air were sampled through two separated thermal desorption tubes using Gilian LFS-113 air pumps (Sensidyne LP). The pumps of stability stream lower than 5 % were used in the examination. Samples were collected over 5 h a day with 4.5 L volume of air passed. The procedure also included checking the rate of air stream flowing through the sorbent tube using a flowmeter. The measuring cycle was continued for each device for four consecutive days.

**Fig. 1:**
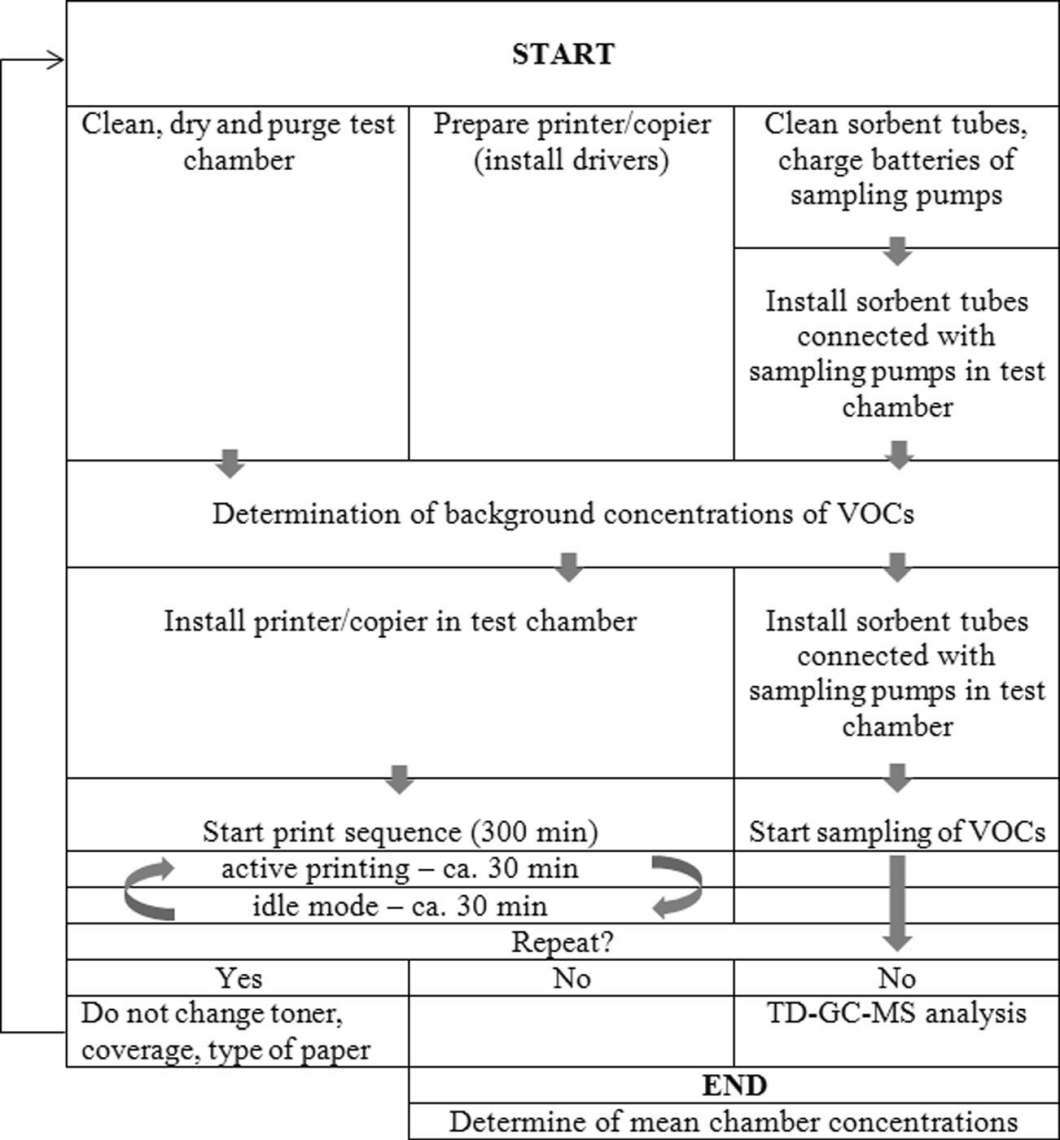
Procedure scheme of measurement of emissions from office printers and copiers

The walls of the test chamber at the end of each measurement day were cleaned with water, then with methanol in order to remove potential chemical substances present on the inner surface of the chamber. The emission test chamber was then dried and purged at test conditions. Zero sample was included in the calculations of the range of emitted substances. The scheme of measurements carried out in order to assess emissions from office printers and copiers for one testing day is presented in Fig. 1.

Stainless steel thermal desorption tubes (Markes International) filled with Tenax (130 mg), Carbograph 1TD (190 mg) and Carboxen 1000 (110 mg) were used for VOCs sampling. Before each measurement, the sorbent tubes were cleaned and conditioned for 15 min at 100, 200, 300 and 335 °C.

### Analytical method

Samples were desorbed by heating them up to 300 °C in a thermal desorber (TD Unity, Markes International). The operation parameters for thermal desorber were as follows: desorption temperature, 300 °C; desorption time, 10 min; flow rate of carrier gas (helium), 33 mL/min; cold trap temperature, −10 °C; cold trap desorption temperature, 300 °C; and desorption time, 3 min.

Analytes were then transferred by inert carrier gas (helium) (99.999 % purity, Multax s.c.) into the gas chromatograph (GC 6890N, Agilent Technologies) and analyzed using the mass selective detector (MSD 5975C, Agilent Technologies). Separation was performed on a capillary column with HP-5MS (J & W Scientific; 30 m × 0.25 mm, i.d. × 0.25 μm film thickness), with the following temperature programme: 40 °C/4 min/5 °C min^−1^/225 °C/0.5 min/15 °C min^−1^/240 °C/5 min. Helium was maintained at a constant flow rate of 1.1 mL/min.

Full mass spectra were taken at the energy of ionizing electrons of 70 eV and in the mass range of 40–450 amu. For substance identification, the mass spectrum library NIST 05 was available.

### Calibration

The calibration of VOCs was conducted by adsorbing the certified VOCs standard solution in methanol with a concentration (of each compound) of 200 μg/mL (EPA 524.2 VOC-20Mix, Supelco) onto multi-bed thermal desorption tubes filled with Tenax, Carbograph 1TD and Carboxen 1000. The calibration gas mixture was prepared in a stainless steel canister (SilcoCan, Restek; 6 L) according to description presented in “Chemicals and standards”.

Volatile organic compound calibration curves (5 points) were determined for samples from 1 ng to 112.5 ng of each compound spread over the sorbent tube. Toluene, benzene, xylenes, ethylbenzene, styrene, naphthalene, butyl acetate, chlorobenzene, 1,2-dichlorobenzene, 1,4-dichlorobenzene, tetrachloroethylene, 1,2,4-trichlorobenzene and trichloroethylene were individually quantified using the relative response factors determined from a standard calibration. Table 1 presents the retention times of volatile organic compounds and validation data of the examination method. The quantification of other substances was performed on the assumption of the response factor of toluene.

**Table 1:** Retention times (RT) of VOCs and validation data of the examination method

VOC	RT [min]	Measurement range [μg m^−3^]	Equation of the calibration curve	Correlation coefficient	Limit of detection [μg m^−3^]	Limit of quantification [μg m^−3^]	Precision [%]
Benzene	2.78	0.4–11	167,225x + 418,483	0.9988	0.01	0.03	5.99
		11–44	197,294x + 157,024	0.9989			
Trichloroethylene	3.27	0.4–11	442,760x + 621,235	0.9999	0.02	0.05	6.84
Toluene	4.58	0.4–11	721,328x + 56,108	0.9999	0.01	0.03	7.32
		11–44	654,444x − 8,413,649	0.9998			
		46.3–370.7	902,973x + 1,861,071	0.9992			
Tetrachloroethylene	5.79	0.4–11	969,296x + 384,238	0.9987	0.007	0.02	5.57
Butyl acetate	6.06	0.46–27.8	239,743x−60,438	0.9994	0.002	0.008	7.82
Chlorobenzene	6.97	0.4–11	526,266x + 668,139	0.9982	0.01	0.04	5.70
Ethylbenzene	7.47	0.4–11	966,381x + 2,083,209	0.9993	0.007	0.02	6.30
		11–44	1,140,611x−2,068,484	0.9996			
*m*-, *p*-Xylene	7.74	0.4–11	1,774,148x + 2,881,217	0.9994	0.01	0.03	7.04
		11–44	1,215,289x + 13,904,661	0.9987			
*o*-Xylene	8.45	0.4–11	1,818,879x + 1,567,847	0.9988	0.007	0.02	5.50
		11–44	1,230,227x + 42,782,952	0.9999			
Styrene	8.59	0.4–11	675,856x + 1,137,873	0.9996	0.01	0.03	6.38
		11–44	715,852x−6,354,713	0.9989			
1,3,5-Trimethylbenzene^a^	11.27	0.4–11	721,328x + 56,108	0.9999	0.01	0.03	7.32
		11–44	654,444x − 8,413,649	0.9998			
α-Methylstyrene^a^	11.80	46.3–370.7	902,973x + 1,861,071	0.9992	0.01	0.03	7.32
1,2,3-Trimethylbenzene^a^	12.17	0.4–11	721,328x + 56,108	0.9999	0.01	0.03	7.32
		11–44	654,444x−8,413,649	0.9998			
1,2,4-Trimethylbenzene^a^	13.20	0.4–11	721,328x + 56,108	0.9999	0.01	0.03	7.32
		11–44	654,444x−8,413,649	0.9998			
1,4-Dichlorobenzene	12.61	0.4–11	989,383x + 1,243,438	0.9998	0.007	0.021	6.42
Butylcyclohexane^a^	13.48	46.3–370.7	902,973x + 1,861,071	0.9992	0.01	0.03	7.32
1,2-Dichlorobenzene	14.17	0.4–11	156,645x + 233,184	0.9999	0.004	0.012	6.13
Decamethylcyclopentasiloxane^a^	17.76	0.4–11	721,328x + 56,108	0.9999	0.01	0.03	7.32
		11–44	654,444x − 8,413,649	0.9998			
		46.3–370.7	902,973x + 1,861,071	0.9992			
1,2,4-Trichlorobenzene	18.37	0.4–11	689,137x + 2,098,565	0.9996	0.01	0.03	5.83
Naphthalene	18.54	0.4–11	1,373,157x + 1,955,718	0.9995	0.005	0.01	5.32
		11–44	1,393,397x−9,016,823	0.9988			

^a^The quantification of these substances was performed on the assumption of the response factor of toluene

### Calculation of results

Mass concentration of the analyte (C) in air samples taken from the test chamber was calculated in micrograms per cubic meter according to the following formula:

1 $$ C=\frac{m_a}{Q\cdot {t}_s}\cdot {10}^6 $$

where:

*C*: is the emission test chamber concentration [μg m^−3^]
*m*_*a*_: is the mass of the analyte adsorbed in the sorbent tube [μg]
*Q*: is the sampling flow rate (flow rate of the air stream through the sorbent tube) [mL/min]
*t*_*s*_: is the sampling time (the time of collecting the air sample) [min]

Average mass concentration was calculated as an arithmetic mean of the samples taken during four measurement days.

The results were expressed by the unit-specific emission rate (*q*_*u*_) in micrograms per unit and hour. Specific unit rate emission was calculated according to the following formula:

2 $$ {q}_u=\frac{m_a\cdot n\cdot {V}_{\mathrm{ch}}\cdot {t}_s}{V_s\cdot {t}_d\cdot u} $$

where:

*q*_*u*_: is the unit-specific emission rate [μg · unit^−1^ · h^−1^]
*m*_*a*_: is the analyzed mass of VOC (the mass of adsorbed analyte in the sorbent tube) [μg]
*n*: is the air change rate [h^−1^]
*V*_ch_: is the chamber volume [m^−3^]
*t*_*s*_: is the sampling time [min]
*V*_*s*_: is the sample volume (the volume of the air through the sorbent tube) [m^−3^]
t_d_: copying/printing time [min]
*u*: number of test products [unit].

The average specific emission (*e*_*p*_), in micrograms per page, was calculated using the formula:

3 $$ {e}_p=\frac{m_a}{p} $$

where *p* is the number of pages [page].

## Results and discussion

### Analysis of VOCs mixture

The performed air pollution measurements showed emissions of multi-component mixtures of chemicals into the space of test chamber from all of the office devices covered by the tests during their operation (printing and copying). Examples of chromatograms of VOCs emitted from devices A and D are reported in the Supplementary material (SM1, SM2). The number of substances identified in the analyzed air reached even more than 60 compounds in some cases. The chemical substances found among the identified factors belong to the group of volatile organic compounds, which included benzene, toluene, chlorobenzene, ethylbenzene, xylenes, styrene, nonane, propylbenzene, trimethylbenzene, 1-methyl-3-propylbenzene, decamethylcyclopentasiloxane, dodecane, 2-phenoxyethanol, tridecane and pentadecane. Some of the compounds were released during printing from all of the appliances covered by the tests (e.g. benzene, toluene, ethylbenzene, xylenes, chlorobenzene and α-pinene), while others occurred sporadically, e.g. butylcyclohexane and 4-methyldecane (emitted only by copier D). The emission of these compounds may be related to the composition of the toners used in office equipment.

**Fig. 2:**
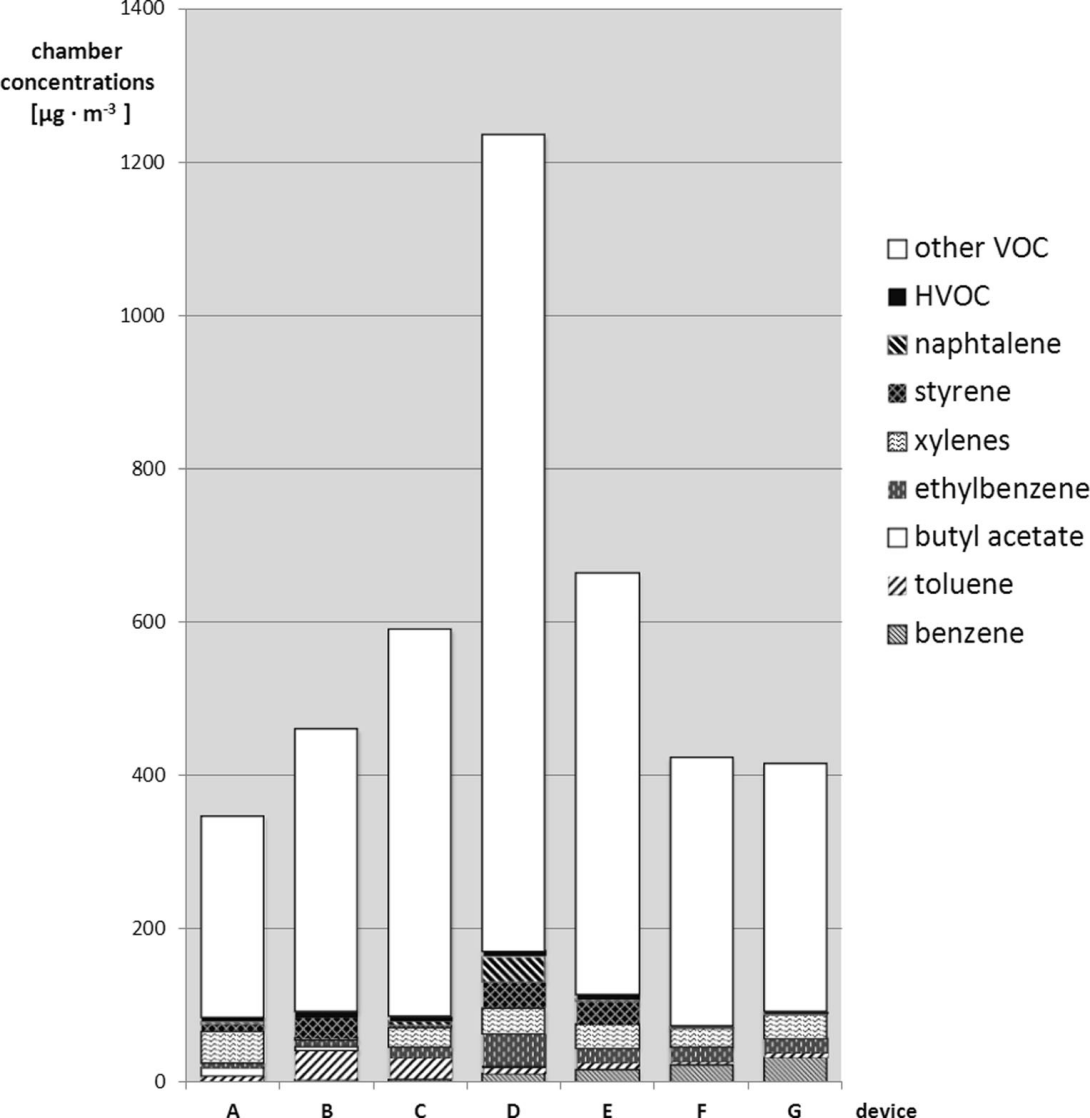
Contribution of the selected volatile organic compounds (VOCs) and halogenated VOCs (HVOC) emitted from the office devices in the total volatile organic compound (TVOC)

Among the determined volatile halogenated compounds, only chlorinated organic compounds were identified: tetrachloroethylene and chlorobenzene (identified while operating all of the appliances) and 1,4-dichlorobenzene (identified in 85 % of samples).

Office printers and copiers are sources of VOCs which derive at least in part from the toner that undergoes heating during the printing processes. During the operation, the VOC emissions were caused by heating up the drum and toner up to 160 °C for compress toner on paper (Ewers and Nowak 2006; Kim et al. 2009). The toners can emit chemical compounds contained therein, as well as the unreacted residue of the produced pigments and the remaining ingredients. The chemical emission printers and copiers can result from circuit boards, inks and toners, papers and plastic construction materials. Kowalska and Gierczak (2013) tested emissions of VOCs and halogenated VOCs of the elements of office equipment. During the tests, at a temperature of 23 °C, samples of plastic chemical compounds such as benzene, toluene, xylenes, chlorobenzene, tetrachloroethylene, trichlorethylene were emitted. Increased temperature (heated enclosure components of devices) may enhance the emission of VOCs (including halogenated VOCs) in the air. As confirmed by other authors (Lee et al. 2001), emissions from printers and copiers also depend on the operation modes as well as the materials used.

### Chamber concentrations

Chromatographic peak areas corresponding to the contents of individual substances obtained after thermal desorption of the triple-layer thermal desorption tubes were used to calculate calibration curves. Mean concentrations of emitted VOCs were at the levels shown in Table 2. All tested devices were characterized by increased levels of decamethylpentasiloxane, trimethylbenzene, xylenes and ethylbenzene.

**Table 2:** Mean concentration values of selected VOCs released from individual office devices

No.	Volatile organic compounds	Mean chamber concentrations in μg m^−3^ (±standard deviation)						
		Device A	Device B	Device C	Device D	Device E	Device F	Device G
1	Benzene	1.15 (±0.14)	3.21 (±0.40)	4.04 (±0.51)	10.99 (±1.4)	16.9 (±2.2)	23.1 (±3.0)	31.7 (±4.1)
2	Trichloroethylene	2.01 (±0.29)	2.00 (±0.29)	1.49 (±0.22)	nd	nd	nd	nd
3	Toluene	7.3 (±1.1)	37.7 (±5.7)	26.6 (±4.0)	7.65 (±1.2)	7.8 (±1.2)	3.53 (±0.55)	5.05 (±0.79)
4	Tetrachloroethylene	0.43 (±0.52)	1.65 (±0.20)	3.89 (±0.47)	2.55 (±0.31)	0.592 (±0.072)	0.245 (±0.030)	0.503 (±0.061)
5	Butyl acetate	10.3 (±1.7)	5.51 (±0.89)	nd	2.48 (±0.40)	nd	nd	nd
6	Chlorobenzene	3.68 (±0.46)	3.83 (±0.48)	0.607 (±0.076)	0.331 (±0.041)	6.48 (±0.81)	1.09 (±0.14)	2.31 (±0.29)
7	Ethylbenzene	7.4 (±1.0)	8.9 (±1.2)	15.1 (±2.0)	41.3 (±5.5)	20.4 (±2.7)	20.01 (±2.7)	20.6 (±2.8)
8	Xylenes	40.5 (±6.2)	2.72 (±0.41)	25.6 (±3.7)	34.0 (±4.9)	29.9 (±4.4)	24.5 (±3.6)	30.7 (±4.5)
9	Styrene	9.7 (±1.3)	26.1 (±3.6)	2.69 (±0.36)	33.7 (±4.5)	30.1 (±4.0)	nd	nd
10	Trimethylbenzene	45.6 (±7.1)	12.5 (±1.9)	24.9 (±3.9)	18.8 (±2.9)	20.78 (±3.2)	16.1 (±2.5)	8.3 (±1.3)
11	α-Methylstyrene	nd	nd	nd	489 (±76)	690 (±110)	106 (±17)	208 (±44)
12	Butylcyclohexane	nd	nd	nd	78 (±12)	nd	nd	nd
13	1,4-Dichlorobenzene	0.233 (±0.032)	0.514 (±0.070)	0.347 (±0.048)	0.702 (±0.097)	nd	0.153 (±0.021)	0.181 (±0.025)
14	1,2-Dichlorobenzene	nd	0.437 (±0.057)	0.085 (±0.012)	nd	nd	nd	nd
15	Decamethylcyclopentasiloxane	33.7 (±5.3)	62.0 (±9.7)	67 (±11)	59.9 (±9.4)	47.4 (±7.4)	55.5 (±8.7)	28.1 (±4.4)
16	1,2,4-Trichlorobenzene	nd	0.0167 (±0.0026)	nd	4.85 (±0.63)	nd	0.110 (±0.014)	nd
17	Naphtalene	2.67 (±0.31)	nd	5.52 (±0.65)	33.0 (±3.7)	2.54 (±0.30)	1.07 (±0.13)	1.15 (±0.14)
18	TVOC	347 (±53)	461 (±71)	591 (±91)	1235 (±190)	660 (±100)	424 (±65)	416 (±64)
19	HVOC	6.35 (±0.80)	8.5 (±1.1)	6.42 (±0.82)	8.4 (±1.1)	7.07 (±0.89)	1.60 (±0.20)	2.99 (±0.37)

*nd* not detected, *TVOC* sum of concentrations of identified and unidentified volatile organic compounds eluting between and including the *n*-hexane and *n*-hexadecane (ISO 16000–9:2006), *HVOC* sum of halogenated volatile organic compounds

The identified compounds included toluene, benzene, xylenes, ethylbenzene and styrene as the compounds that pose hazard to human health when present in air. Higher mass concentrations were noted for xylenes up to 40.5 μg m^−3^, ethylbenzene up to 41.3 μg m^−3^, trimethylbenzene up to 45.6 μg m^−3^ and for toluene up to 37.7 μg m^−3^.

The concentrations of aromatic hydrocarbons obtained in this study were similar with those reported by Smola et al. (2002) for benzene, toluene, ethylbenzene, xylenes and styrene. This indicates that office equipment (printers, copiers) of a new type, although probably of different design and enhanced features of print, can still emit dangerous chemical compounds.

During the examination of VOC emission of selected office devices, the following halogenated organics were marked in the chamber: chlorobenzene, 1,2-dichlorobenzene, 1,4-dichlorobenzene, tetrachloroethylene, 1,2,4-trichlorobenzene and trichloroethylene. All of the abovementioned compounds were identified while printing with the use of office laser printer B. The highest amount of chlorobenzene (average concentration 6.48 μg m^−3^) was emitted by copier E (Tab. 2), which was also the source of the emission of tetrachloroethylene (average concentration 0.59 μg m^−3^).

There is little information about halogenated volatile organic compounds emitted from office devices in available literature. Lee et al. (2001) determined the concentration of chloroorganic compounds, i.e. chloromethane, dichloromethane and trichloromethane, while printing with three printers (two lasers and one inkjet). Tetrachloroethylene, 1, 2-, 1,3- and 1,4-dichlorobenzene and 1,4-dichlorobenzene were marked during tests of inkjet printer and copier only. The maximum concentration of tetrachloroethylene (up to 3.89 μg m^−3^) and 1,2,4-trichlorobenzene (up to 4.85 μg m^−3^) obtained in this work are comparable with the results of Lee et al. (up to 2.92 and 4.75 μg m^−3^ accordingly). Maximum concentration of 1,2-dichlorobenzene and 1,4-dichlorobenzene (0.44 and 0.70 μg m^−3^ accordingly) were about three times lower than in the publication from 2001 (1.20 and 1.26 μg m^−3^ for 1,2-dichlorobenzene, 1.92 and 2.10 μg m^−3^ for 1,4-dichlorobenzene) (Lee et al. 2001). This reflects a diverse halogenated organic compound emission from office equipment. The obtained results confirm the necessity of monitoring the air quality in working spaces to improve the safety of workers.

The only halogenoorganic compound identified when examining 16 office devices by Wilke et al. (2009) (and in case of only one device) was trichloroethene. The average concentration value of trichloroethylene emitted was 8 μg m^−3^, which was about four times higher than the concentration calculated for printers A, B and C (2.01, 2.00, 1.49 μg m^−3^ accordingly) in the presented study.

Among emitted VOCs, there were chemical compounds listed in the Regulation of the Minister of Health and Social Welfare of 12 March 1996 (Official Gazette of the Republic of Poland no. 19, item 231, 1996). These compounds were benzene, toluene, butyl acetate, chlorobenzene, ethylbenzene, xylene, styrene, naphthalene, dichlorobenzene and trichloroethylene (Fig. 2). Table 3 presents basic statistics for VOC concentration levels: minimum, maximum and mean values of VOCs emitted from office printers and copiers. Ratios of the mean values of the VOCs’ concentrations (R) were determined. If the specified range of calculated ratio ± uncertainty (R ± U) contains the value 1, there was no statistically significant difference compared to the mean values. Data presented in Table 3 did not show statistically significant difference in emission of most VOCs from tested office printers and copiers.

**Table 3:** Statistical analysis of the chamber concentrations of VOCs

VOC	Chamber concentrations (office printers A–C) [μg m^−3^]				Chamber concentrations (office copiers D–F) [μg m^−3^]				R ± U
	Min	Max	Mean_p_	s_p_	Min	Max	Mean_c_	s_c_	
Benzene	1.15	4.04	2.8	1.49	10.99	31.7	20.7	8.86	7.38 ± 1.53
Trichloroethylene	1.49	2.01	1.83	0.297	–	–	–	–	–
Toluene	7.3	37.7	23.9	15.4	3.53	7.8	6.01	2.08	3.97 ± 2.08
Tetrachloroethylene	0.43	3.89	1.99	1.75	0.245	2.55	0.973	1.06	2.05 ± 2.77
Butyl acetate	5.51	10.3	7.91	3.39	2.48	2.48	2.48	0	3.19 ± 1.30
Chlorobenzene	0.607	3.83	2.71	1.82	0.331	6.48	2.55	2.745	0.94 ± 2.50
Ethylbenzene	7.4	15.1	10.5	4.08	20.01	41.3	25.6	10.5	2.44 ± 1.25
Xylenes	2.72	40.5	22.9	19.0	24.5	34	29.8	3.94	1.30 ± 1.47
Styrene	2.69	26.1	12.8	12.0	30.1	33.7	31.9	2.55	2.49 ± 1.10
Trimethylbenzene	12.5	45.6	27.7	16.7	8.3	20.78	16.0	5.48	1.73 ± 1.61
α-Methylstyrene	–	–	–	–	106	690	373	266	–
Butylcyclohexane	–	–	–	–	78	78	78	0	–
1,4-Dichlorobenzene	0.233	0.514	0.365	0.141	0.153	0.702	0.345	0.3092	1.06 ± 1.92
1,2-Dichlorobenzene	0.085	0.437	0.261	0.249	–	–	–	–	–
Decamethylcyclopenta-siloxane	33.7	67	54.2	17.9	28.1	59.9	47.7	14.1	1.14 ± 0.89
1,2,4-Trichlorobenzene	0.0167	0.0167	0.0167	–	0.11	4.85	2.48	3.35	148.50 ± 5.37
Naphthalene	2.67	5.52	4.10	2.02	1.07	33	9.44	15.7	2.31 ± 4.68
TVOC	347	591	466	122	416	1235	684	385	1.47 ± 1.40
HVOC	6.35	8.5	7.09	1.22	1.6	8.4	5.02	3.24	1.41 ± 1.14

*U* uncertainty for the calculated ratio (R) of obtained mean values: = $k\frac{\sqrt{\left({s}_p^2+{s}_c^2\right)}}{\left(\frac{{\mathrm{mean}}_p^2+{\mathrm{mean}}_c^2}{2}\right)}$ where coverage factor (*k* = 2 for the level of confidence of 95 %), *s* standard deviation, *TVOC* sum of concentrations of identified and unidentified volatile organic compounds eluting between and including the *n*-hexane and *n*-hexadecane (ISO 16000–9:2006), *HVOC* sum of halogenated volatile organic compounds

**Fig. 3:**
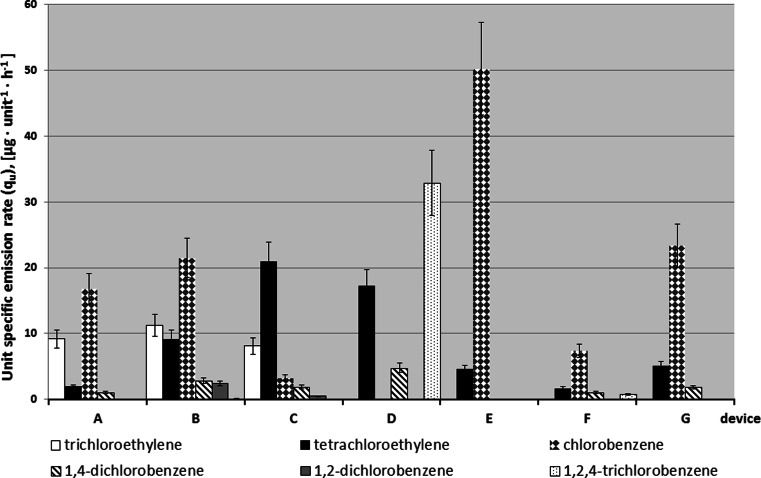
The values of specific unit emission rate of halogenated volatile organic compounds emitted by particular office devices

Due to the variety of volatile organic compounds (VOCs) occurring in the air inside the chamber, quantities of emissions from the devices for tested air samples were based on a calculated sum of the volatile compounds emitted (TVOC) (ISO 16000–9 2006). The TVOC value is specific for the tested product and used for comparing products with a similar target VOCs emission profile. The average value of TVOC for particular printing devices (A–C) were up to 591 μg m^−3^ and for copying devices (D–G) up to 1235 μg m^−3^. Comparison of the mean TVOC values obtained for all measuring days revealed that the highest concentration of these compounds in the air occurred while copying with the device D. The highest contribution in TVOC value had inter alia α-methylstyrene, butylcyclohexane and decamethylcyclopentasiloxane (labeled as *other VOC*) (Fig. 3).

α-Methylstyrene was the most numerous compound emitted from copying devices. Some studies show that ethylbenzene, styrene and α-methylstyrene are included in the toner (among other VOCs) (Kagi et al. 2007; Ewers and Nowak 2006). Salthammer et al. (2012) suggested that the identified polisiloxanes came from technical mixtures of silicone oil or silicone grease which was used as heat-resistant lubricants in printing devices. Aromatic compounds were among the most ubiquitous VOCs detected in indoor air (Wang et al. 2011; Kagi et al. 2007; Lee et al. 2001; Smola et al. 2002).

Comparable TVOC results, presented by the authors of this work, were obtained by Brown (1999) during copying one-page documents (i.e. 370, 570 and 900 μg m^−3^). The TVOC results obtained in this work were higher than the calculated sum of four printers examined by Tuomi et al. (2000) (90–170 μg m^−3^), but were within the range of 29–3287 μg m^−3^ obtained after the evaluation of 16 office devices by Wilke et al. (2009).

Considering the total mass concentration of halogenated volatile organic compounds (HVOC), their largest sources were device B (laser printer) (8.45 μg m^−3^; which constituted 1.83 % of the emitted TVOC) and device D (copier) (8.43 μg m^−3^; which constituted 0.68 % of the emitted TVOC). The contribution of HVOC in the total emissions of VOCs from tested office devices was minor.

### Specific emission rate

Figure 3 shows calculated unit-specific emission rate q_u_ (in micrograms per unit and hour) of halogenated volatile organic compounds of office devices A–C (office printers) and D–G (office copiers). Device E emitted only two halogenated compounds, while other devices (B and C) discharged mixtures of six and five halogenated organic compounds. The halogenated VOCs which had higher unit emission rates were chlorobenzene (50.2 μg unit^−1^ h^−1^) and 1,2,4-trichlorobenzene (32.9 μg unit^−1^ h^−1^). Arranged in order from the biggest to the smallest value of specific unit emission rate, the following sequences were obtained:

- D>E>G>C>F>B>A for unit-specific emission rate of TVOC
- D>E>B>C>G>A>F for unit-specific emission rate of HVOC

Copiers D and E were the biggest sources of emission of volatile organic compounds (including halogenated compounds). The value of unit-specific emission rate of TVOC obtained in this work (from 1585 to 8382 μg unit^−1^ h^−1^) were higher than the results obtained by Tuomi et al. (2000) (i.e. 600–1200 μg unit^−1^ h^−1^for four printers).

The size of q_u,TVOC_ and q_u,HVOC_ did not depend on the exploitation time of the device. Device A (manufactured in 1998) was not a greater source of VOCs emission than the devices produced later. Printing speeds declared by the producers were highest for copiers D, E and G (more than 15 page/min). High values of q_u_ determined for these devices results from larger number of pages that have been printed at the test time.

Table 4 shows determined specific emission calculated per one printed page (in micrograms per page) of volatile organic compounds. As compared with the results of seven laser office printers presented by Brown (2011), the office devices (A–G) examined in this work emitted twice more (in case of xylenes) to over six times as many (in case of styrene) micrograms of volatile organic compounds calculated per one printed page. Brown (2011) did not determine their halogenated derivatives among emitted organic compounds.

**Table 4:** The values of specific emission calculated per one printed page of particular office devices

No.	VOC	Average specific emission (e_*p*_) in μg page^−1^ (±standard deviation)						
		Device						
		A	B	C	D	E	F	G
1	Benzene	0.04 (±0.004)	0.10 (±0.01)	0.20 (±0.02)	0.24 (±0.03)	0.38 (±0.05)	0.83 (±0.11)	0.92 (±0.12)
2	Trichloroethylene	0.07 (±0.01)	0.06 (±0.01)	0.08 (±0.01)	nd	nd	nd	nd
3	Toluene	0.24 (±0.04)	1.12 (±0.17)	1.34 (±0.20)	0.17 (±0.02)	0.17 (±0.02)	0.13 (±0.02)	0.15 (±0.02)
4	Tetrachloroethylene	0.01 (±0.001)	0.05 (±0.006)	0.20 (±0.02)	0.06 (±0.01)	0.01 (±0.001)	0.01 (±0.001)	0.02 (±0.002)
5	Butyl acetate	0.34 (±0.06)	0.16 (±0.03)	nd	0.05 (±0.01)	nd	nd	nd
6	Chlorobenzene	0.12 (±0.02)	0.11 (±0.01)	0.03 (±0.004)	0.01 (±0.001)	0.15 (±0.02)	0.04 (±0.005)	0.07 (±0.01)
7	Ethylbenzene	0.25 (±0.03)	0.26 (±0.03)	0.76 (±0.10)	0.89 (±0.12)	0.45 (±0.06)	0.72 (±0.10)	0.60 (±0.08)
8	Xylenes	1.35 (±0.21)	0.08 (±0.01)	1.29 (±0.20)	0.73 (±0.11)	0.67 (±0.10)	0.88 (±0.13)	0.90 (±0.13)
9	Styrene	0.32 (±0.04)	0.78 (±0.11)	0.14 (±0.02)	0.73 (±0.10)	0.67 (±0.09)	nd	nd
10	Trimethylbenzene	1.52 (±0.27)	0.37 (±0.07)	1.25 (±0.22)	0.41 (±0.08)	0.46 (±0.08)	0.58 (±0.11)	0.24 (90.04)
11	α-Methylstyrene	nd	nd	nd	10.57 (±2.11)	15.46 (±3.1)	3.80 (±0.76)	6.07 (±1.21)
12	Butylcyclohexane	nd	nd	nd	1.69 (±0.27)	nd	nd	nd
13	1,4-Dichlorobenzene	0.01 (±0.001)	0.02 (±0.003)	0.02 (±0.003)	0.02 (±0.003)	nd	0.005 (±0.0007)	0.005 (±0.0007)
14	1,2-Dichlorobenzene	nd	0.01 (±0.001)	0.005 (±0.008)	nd	nd	nd	nd
15	Decamethylcyclopentasiloxane	1.12 (±0.21)	1.85 (±0.35)	3.38 (±0.64)	1.29 (±0.24)	1.06 (±0.20)	1.99 (±0.38)	0.82 (±0.15)
16	1,2,4-Trichlorobenzene	nd	0.001 (±0.0001)	nd	0.11 (±0.01)	nd	0.004 (±0.0005)	nd
17	Naphthalene	0.09 (±0.01)	nd	0.28 (±0.03)	0.71 (±0.09)	0.06 (±0.007)	0.04 (±0.005)	0.03 (±0.004)
18	TVOC	11.55 (±1.85)	13.71 (±2.19)	29.71 (±4.75)	26.67 (±4.27)	14.81 (±2.37)	15.17 (±2.43)	12.10 (±1.95)
19	HVOC	0.21 (±0.03)	0.25 (±0.04)	0.32 (±0.05)	0.18 (±0.03)	0.16 (±0.03)	0.06 (±0.01)	0.09 (±0.02)

*nd* not detected, *TVOC* sum of concentrations of identified and unidentified volatile organic compounds eluting between and including the *n*-hexane and *n*-hexadecane (ISO 16000–9:2006), *HVOC* sum of halogenated volatile organic compounds

Comparing values of average specific emission _(_ *e*_*p*)_ calculated according to formula (3), the biggest source of VOCs and halogenated compounds was inkjet printer (device C). This is understandable if taking into account the printing speed. Inkjet printer (device C) was the slowest printing device, and results of the compounds emission concentration in the chamber and unit-specific emission rates were quite high.

## Conclusions

The obtained results added more evidence on the importance of monitoring the air quality in working spaces to improve the safety of workers. Tested office printers and copiers emit chemical substances that are harmful to health, including benzene and trichloroethylene, which are classified according to IARC to group 1 (carcinogenic factors for people).

The comparison among seven office devices showed that all of them emit VOCs although with differences in individual compounds and their concentrations. Higher mass concentrations were noted for xylenes up to 40.5 μg m^−3^, ethylbenzene up to 41.3 μg m^−3^, trimethylbenzene up to 45.6 μg m^−3^ and toluene up to 37.7 μg m^−3^. Among VOCs emitted to the air in test chambers, halogenated organic compounds (up to 2 % of the emitted VOCs), i.e. chlorobenzene, 1,2-dichlorobenzene, 1,4-dichlorobenzene, tetrachloroethylene, 1,2,4-trichlorobenzene and trichloroethylene, were determined.

The office devices may be significant sources of chemical emission in small environments. Therefore, the organization of work in office should take into account the placement of printers and copiers as far as possible from the desks (workplaces) in a place with adequate ventilation.

The thermal desorption method combined with gas chromatography – mass spectrometry may be used for the identification and simultaneous quantitative determination of traces of VOCs emitted by printing and copying devices.

## Electronic supplementary material

Below is the link to the electronic supplementary material.

Supplementary Material 1 GC-MS chromatogram of VOCs emitted into the air in the test chamber by operating device A (GIF 255 kb)

High resolution image (TIFF 1701 kb)

Supplementary Material 2 GC-MS chromatogram of VOCs emitted into the air in the test chamber by operating device D (GIF 276 kb)

High resolution image (TIFF 1700 kb)
