# Supplementary figures and images for: Measurements of chlorinated volatile organic compounds emitted from office printers and photocopiers
Source: Environ Sci Pollut Res Int. 2014 Oct 18;22(7):5241–52. doi: 10.1007/s11356-014-3672-3 (PMC4366558; doi:10.1007/s11356-014-3672-3)

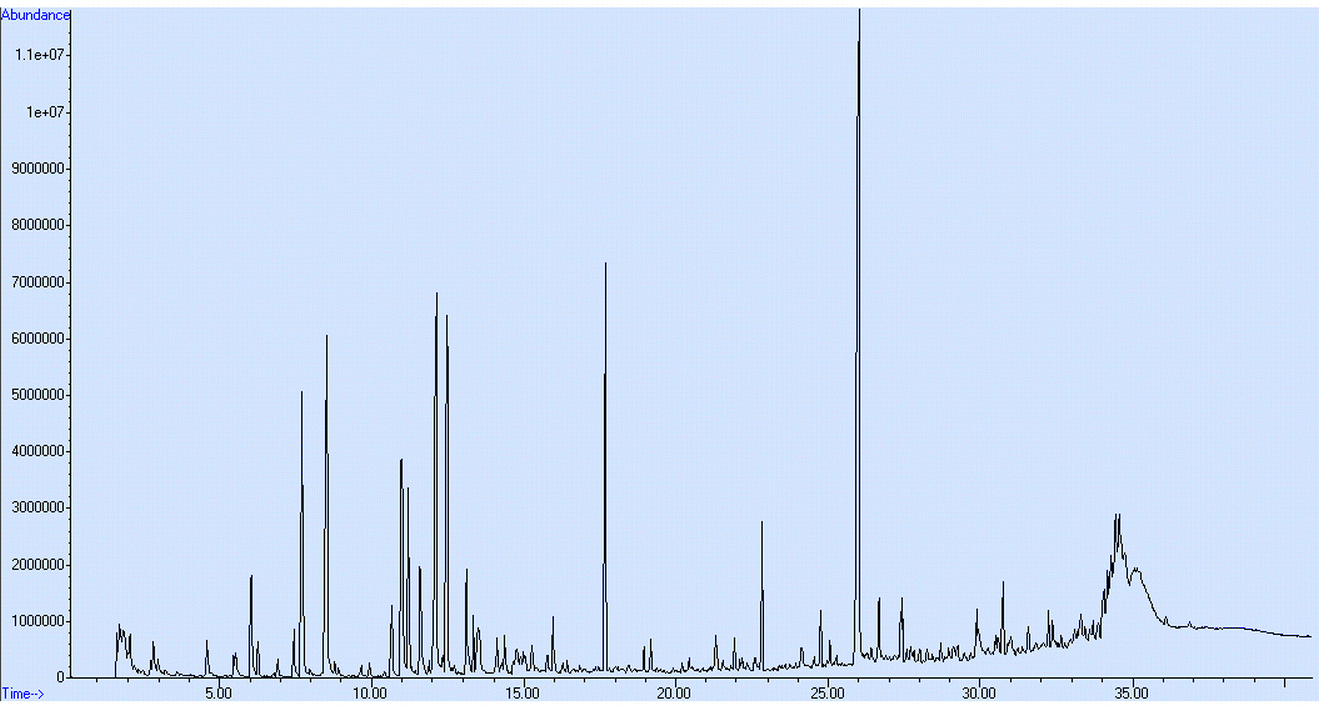

Supplement: Supplementary file 1 — GC-MS chromatogram of VOCs emitted into the air in the test chamber by operating device A (GIF 255 kb) [file 11356_2014_3672_Fig4_ESM.gif]

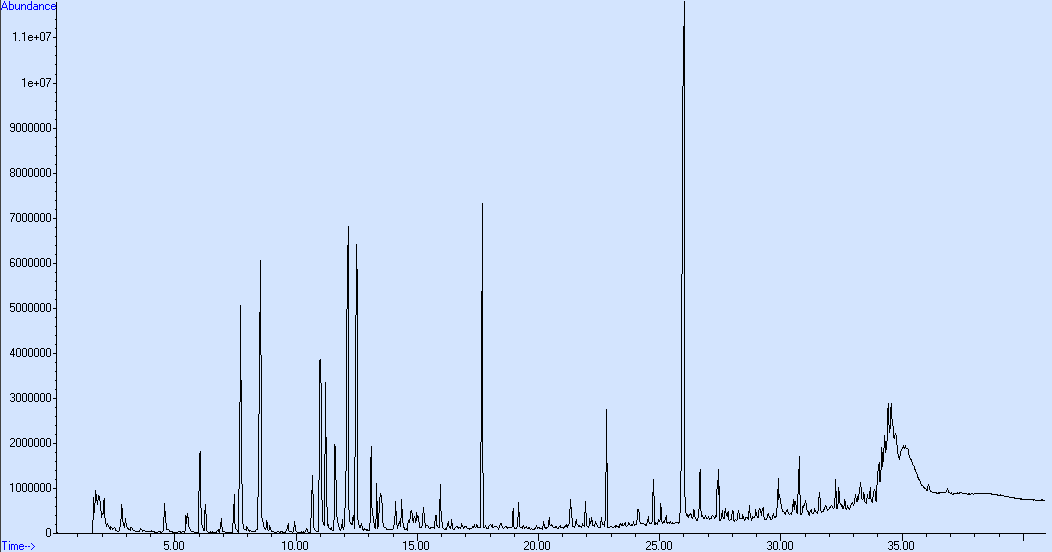

Supplement: Supplementary file 2 — High resolution image (TIFF 1701 kb) [file 11356_2014_3672_MOESM1_ESM.tiff]

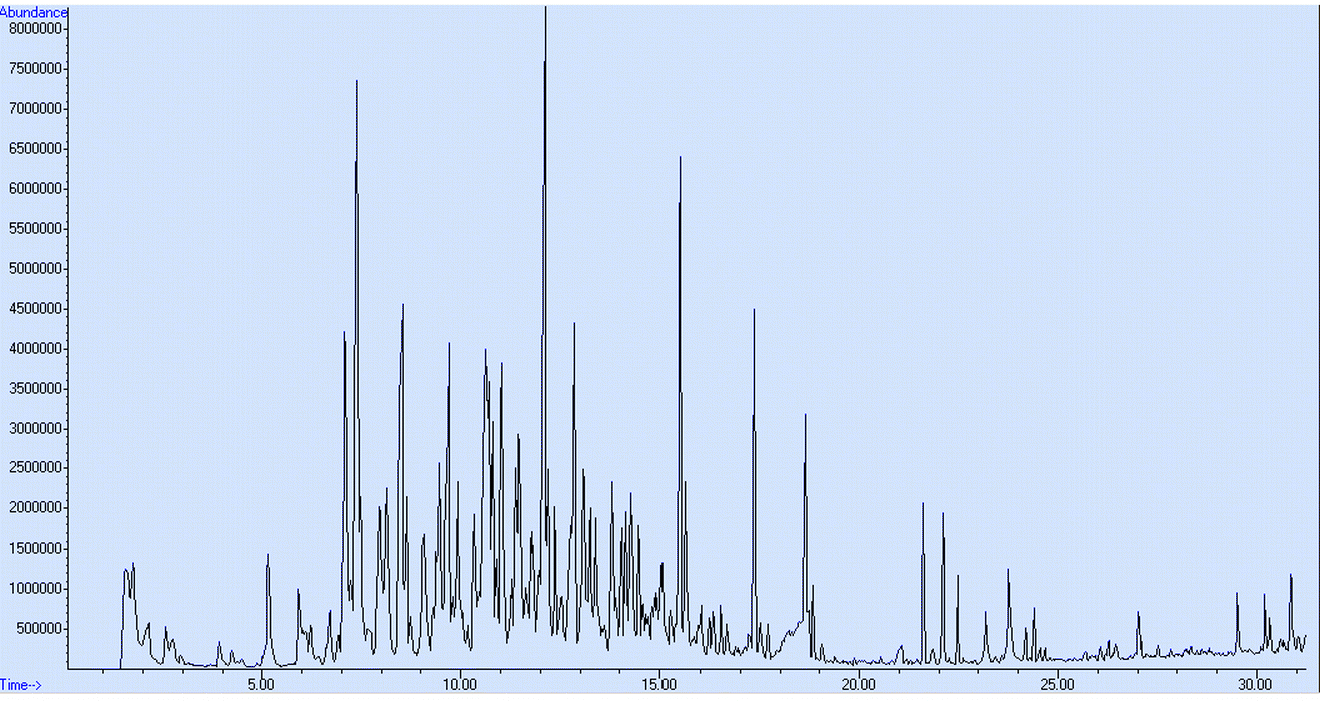

Supplement: Supplementary file 3 — GC-MS chromatogram of VOCs emitted into the air in the test chamber by operating device D (GIF 276 kb) [file 11356_2014_3672_Fig5_ESM.gif]

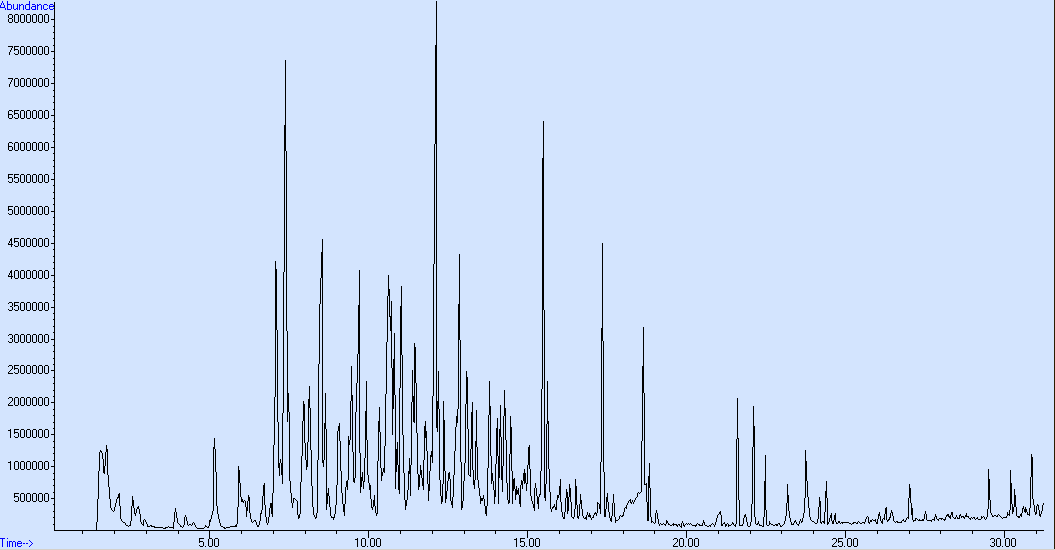

Supplement: Supplementary file 4 — High resolution image (TIFF 1700 kb) [file 11356_2014_3672_MOESM2_ESM.tiff]
